# Long Noncoding RNAs in Taxane Resistance of Breast Cancer

**DOI:** 10.3390/ijms241512253

**Published:** 2023-07-31

**Authors:** Hailong Chen, Mengwen Zhang, Yongchuan Deng

**Affiliations:** 1 Department of Breast Surgery, The Second Affiliated Hospital, Zhejiang University School of Medicine, Hangzhou 310009, China; enhail@zju.edu.cn; 2 Department of Plastic Surgery, The Second Affiliated Hospital, Zhejiang University School of Medicine, Hangzhou 310009, China; zhangmw@zju.edu.cn

**Keywords:** breast cancer, long noncoding RNAs, taxane resistance

## Abstract

Breast cancer is a common cancer in women and a leading cause of mortality. With the early diagnosis and development of therapeutic drugs, the prognosis of breast cancer has markedly improved. Chemotherapy is one of the predominant strategies for the treatment of breast cancer. Taxanes, including paclitaxel and docetaxel, are widely used in the treatment of breast cancer and remarkably decrease the risk of death and recurrence. However, taxane resistance caused by multiple factors significantly impacts the effect of the drug and leads to poor prognosis. Long noncoding RNAs (lncRNAs) have been shown to play a significant role in critical cellular processes, and a number of studies have illustrated that lncRNAs play vital roles in taxane resistance. In this review, we systematically summarize the mechanisms of taxane resistance in breast cancer and the functions of lncRNAs in taxane resistance in breast cancer. The findings provide insight into the role of lncRNAs in taxane resistance and suggest that lncRNAs may be used to develop therapeutic targets to prevent or reverse taxane resistance in patients with breast cancer.

## 1. Introduction

Breast cancer is the most frequently diagnosed cancer in women and the leading cause of death worldwide. According to GLOBOCAN 2020, breast cancer was the most commonly diagnosed cancer, with an estimated 2.3 million newly diagnosed cases [1]. With early diagnosis and development of therapeutic drugs, the prognosis of breast cancer has markedly improved. Chemotherapy, endocrine therapy, HER2 (human epidermal growth factor receptor 2)-targeted therapy, and immunotherapy have been applied for diverse breast cancers. However, drug resistance remains an important factor in the poor prognosis of patients with breast cancer.

As an important treatment strategy for breast cancer, chemotherapy is widely used in the treatment of breast cancer and remarkably decreases the risk of death and recurrence. Taxanes, including paclitaxel and docetaxel, are basic chemotherapeutic agents used to treat patients with breast cancer. Although taxanes suppress tumor growth and improve survival in advanced breast cancer, the development of resistance is inevitable and results in treatment failure. However, the mechanisms of taxane resistance are not fully understood, and further investigation into taxane resistance mechanisms is necessary to identify potential biomarkers and develop novel treatments aimed at overcoming taxane resistance.

Noncoding RNAs (ncRNAs) are a class of transcripts that are encoded by the genome but are mostly not translated into proteins. NcRNAs have been found to be dysregulated in various cancers and linked to cancer development [2,3,4,5]. LncRNAs are important members of the ncRNA family that are longer than 200 nucleotides and have been shown to play a significant role in critical cellular processes, such as transcription, translation, epigenetic control, stem cell differentiation, autophagy, and apoptosis. As lncRNAs have been implicated in chemotherapy resistance [6,7,8,9], exploring their role in taxane resistance has become a hot research topic in recent years. This review focuses on the mechanisms of taxane resistance in breast cancer and the role of lncRNAs in the development of taxane resistance.

## 2. Mechanisms of Taxane Resistance in Breast Cancer

The taxane class is a series of derivatives synthesized by isolating the active antitumor component from plants and structurally modifying the active component obtained. Taxanes mainly include paclitaxel, docetaxel, and derivatives with a paclitaxel backbone structure. Paclitaxel is a taxane diterpene isolated from the bark of *Taxus brevifolia* [10,11,12]. Docetaxel is a semisynthetic product of precursors extracted from *Taxus baccata* L. that is structurally similar to paclitaxel [13]. Taxanes have been used to treat certain types of cancers. In particular, the use of taxanes in breast cancer is an important breakthrough that has greatly improved the prognosis of patients with breast cancer [14,15].

By binding to a hydrophobic cleft in β-tubulin [16], paclitaxel and docetaxel affect the dynamic balance between α- and β-tubulin dimers and microtubules, promoting the assembly of tubulin into microtubules and facilitating microtubule polymerization, blocking their depolymerization into subunits, causing cells to arrest in the G2 and M phases, and leading to abnormal mitosis or cessation of cell division, ultimately causing cell death [17,18,19]. Taxane-induced microtubule stabilization causes Bcl-2 phosphorylation, triggering a cascade of events leading to apoptosis [20].

Chemotherapy resistance is a major cause of cancer treatment failure, resulting in death in over 90% of patients with metastatic cancer [21]. As one of the standard strategies for breast cancer treatment, taxane resistance remains a major obstacle affecting prognosis. Therefore, understanding the mechanism of taxane resistance will help to identify biomarkers and develop new therapeutic approaches to overcome taxane resistance in breast cancer.

**Figure 1:**
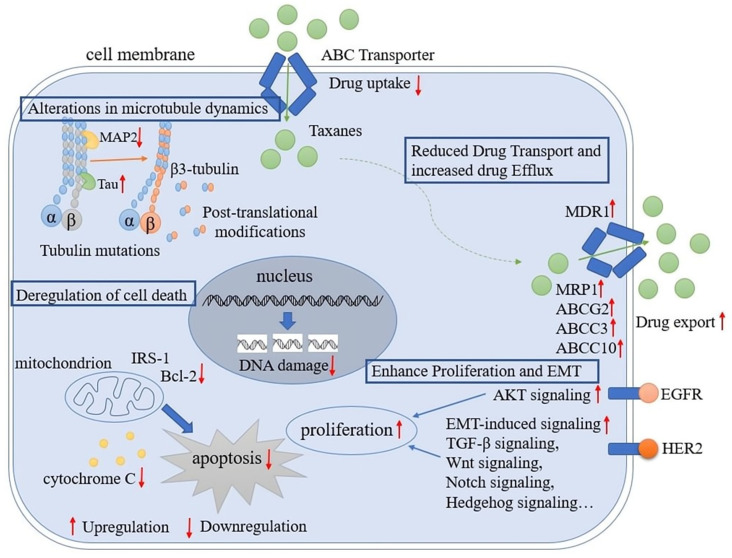
Molecular mechanisms of taxane resistance in breast cancer. The diagram illustrates some of the major mechanisms that are known to contribute to taxane resistance in breast cancer. Upregulated tubulin isotypes and mutations, changes in MAPs, and drug export transporters have been associated with reduced taxane efficacy. Furthermore, upregulated antiapoptotic proteins, upregulated proliferation signaling pathways, and EMT have been linked with increased proliferation after taxane treatment.

The major mechanisms that mediate taxane resistance include (1) alteration of tubulin isotypes and mutations; (2) changes in microtubule-associated proteins (MAPs); (3) drug transport and efflux; (4) deregulation of cell death; (5) alterations in proliferation signaling pathways and the epithelial-to-mesenchymal transition (EMT) (Figure 1).

### 2.1. Alteration of Tubulin Isotypes and Mutations

As mentioned above, microtubules are the targets of taxanes. Alterations in microtubule dynamics and paclitaxel-binding sites on microtubules result in taxane resistance in many cancers [22,23]. Tubulin is the major structural subunit of microtubules: α- and β-tubulin monomers form a dimer that binds to GTP and assembles onto the positive ends of the growing microtubules [24]. α- and β-tubulin (proteins of -450 amino acids each) are highly homologous [25,26], and microtubules are composed of at least eight isotypes of α-tubulin and eight isotypes of β-tubulin [27,28]. Tubulin isotypes can determine the mechanical features of microtubules and control microtubule polymerization dynamics [29]. External stimuli, such as temperature changes or drug exposure, may induce alteration of expression patterns for tubulin isotypes and in this way affect the overall kinetics of microtubule assembly and disassembly [30].

Expression of β-tubulin isotypes plays a key role in many taxane-resistant breast cancers. In particular, microtubules composed of the purified αβ3-tubulin isotype are more dynamic than those composed of αβ2 or αβ4-tubulin isotypes [31] and exhibit a 7.4-fold lower sensitivity to paclitaxel than microtubules assembled from unfractionated tubulin [32]. Kamath et al. reported that β3-tubulin reduces the ability of taxane to suppress microtubule dynamics in cells in the presence of paclitaxel [33]. Furthermore, high levels of β3-tubulin expression are significantly associated with resistance to taxanes in breast cancer [34,35,36,37,38], and treatment with antisense oligonucleotides specific for β3-tubulin can increase paclitaxel efficacy in other cancer cells [33,39]. Some studies have reported that other β-tubulin isotypes (β1-tubulin, β2-tubulin) are also significantly associated with docetaxel resistance in breast cancer [40,41].

In addition to tubulin isotypes, tubulin mutations may be a factor in taxane resistance. The majority of mutational studies have focused on β-tubulin. Although somewhat controversial [30,42], previous studies have shown that mutations in β-tubulin can affect paclitaxel binding and microtubule dynamics [30,43]. Yin et al. suggested that β1-tubulin mutations A185T, A248V, and R306C disrupt microtubule assembly and cause paclitaxel resistance. According to these studies, human tumor cells may develop spontaneous mutations in β1-tubulin, which can lead to resistance to paclitaxel. This implies that patients with certain polymorphisms in β1-tubulin might need higher drug concentrations for effective therapy [44].

Some mutations in β1-tubulin have also been identified in taxane-resistant tumors, e.g., the mutations F270V and A364T in paclitaxel-resistant ovarian cancer cells [45] and the mutation F270L in docetaxel-resistant prostate cancer cells [46]. Therefore, mutation of the β1-tubulin gene has been reported to be one of the mechanisms that causes resistance to taxanes. However, there is not enough evidence to confirm that mutation of β1-tubulin plays an important role in the mechanism of paclitaxel resistance in breast cancer [47].

Microtubules are subjected to numerous posttranslational modifications that may alter their structure and function, including affecting drug resistance. Higher levels of polyglutamylated and acetylated tubulin were observed in paclitaxel-resistant breast cancer cells (MDATXVP) than in sensitive cells [48]. Banerjee et al. reported that paclitaxel-resistant MCF-7 cells contain 2-fold higher amounts of tyrosinated α-tubulin than wild-type MCF-7 cells [49], which demonstrates that paclitaxel resistance is associated with an increase in the level of tyrosinated α-tubulin. Moreover, posttranslational modifications of tubulin have also been shown to be associated with taxane resistance in other types of tumors [50,51]. These modifications affect not only microtubule dynamics but also microtubule organization and interactions with other cellular components.

### 2.2. Changes in Microtubule-Associated Proteins (MAPs)

MAPs represent a significant amount of proteins that regulate microtubule dynamics by interacting with microtubules [52,53,54]. Dysregulation of microtubule dynamics by MAPs may result in resistance to microtubule-targeting drugs, and several studies have demonstrated a correlation between aberrant expression of MAPs and sensitivity to paclitaxel [19].

The MAP2/Tau proteins (including MAP2, MAP4, and Tau) bind along the length of microtubules and stabilize them by altering dynamic behavior, thereby affecting sensitivity to taxanes [55]. Overexpression of MAP2 has been observed in a number of different types of cancer. MAP2 promotes tubulin polymerization and stabilizes microtubules. Elevation of MAP2 in breast cancer cell lines leads to increased paclitaxel sensitivity, and gene expression analysis revealed that MAP2 had significantly higher levels of expression in patients achieving a pathologic complete response to neoadjuvant paclitaxel with radiation treatment [56]. These results indicate the potential use of MAP2 as a tumor marker associated with the response to neoadjuvant taxane-based therapy.

MAP4 is ubiquitously found in all cell types, and the activity of MAP4 in the cell is regulated by phosphorylation [57]. Downregulation or inactivation of MAP4 can increase the dynamics of microtubules and therefore have an impact on paclitaxel resistance [58]. Previous studies have shown that MAP4 is negatively regulated by p53 in the C127 mammary cell line, resulting in increased microtubule polymerization and increased taxane binding and sensitivity [59]. In light of this information, p53-mediated downregulation of MAP4 is a potential mechanism of resistance to taxanes.

Tau has been identified as a potential marker of taxane response in breast cancer [60,61,62,63]. Tau also binds to and stabilizes microtubules. Low levels of tau expression were associated with a greater response to treatment when patients with breast cancer received combinatorial treatment with paclitaxel, 5-fluorouracil, doxorubicin, and cyclophosphamide (P/FAC) [60], and high tau expression showed a significant association with poor response to paclitaxel and trastuzumab chemotherapy in patients with HER2-positive advanced breast cancer [64]. In another study of estrogen receptor (ER)-positive breast cancer patients, high tau mRNA expression indicated tamoxifen sensitivity but not taxane resistance. However, low tau mRNA expression indicates poor prognosis with tamoxifen alone, and patients with low expression may benefit from taxane-containing chemotherapy [65]. However, previous research also showed that neither overexpression nor depletion of tau modulates cellular sensitivity to taxanes and that it is unlikely to be a direct mechanism of taxane resistance in breast cancer [62], perhaps because binding between MAPs and microtubules is regulated by phosphorylation and other factors [55].

Stathmin is a microtubule-destabilizing phosphoprotein that alters cellular microtubule dynamics during interphase and mitosis [54,66]. Previous studies have shown that expression of stathmin is substantially increased in taxane-resistant breast cancer, thereby conferring increased resistance to taxane and an unfavorable prognosis [19,67,68,69]. Moreover, low expression of stathmin predicts a high response to neoadjuvant chemotherapy with docetaxel-containing regimens in locally advanced breast cancer [70]. In the absence of an additional polymerization promoter, intrinsic paclitaxel polymerizing activity is inhibited in the presence of stathmin [71]. Conversely, functional knockdown of stathmin using siRNA results in increased microtubule polymerization, which increases the efficacy of paclitaxel [67].

### 2.3. Drug Transport and Efflux

Drug efflux by transporters is another important mechanism of drug resistance; it relates to the expression of the ATP-dependent transporter family, also known as the ATP-binding cassette (ABC) family, which is composed of at least 48 genes belonging to 7 subfamilies (ABCA through ABCG) [72,73].

P-glycoprotein (P-gp), also known as multidrug resistance1 (MDR1) and ATP-binding cassette subfamily B member 1 (ABCB1), is the most important ABC transporter and is encoded by the MDR-1 gene, which is present on chromosome 7 [72,74]. P-gp is highly expressed in the cell membrane and acts as a multidrug efflux pump that is driven by ATP hydrolysis [75]. Overexpression of P-gp significantly reduces the intracellular concentration of P-gp substrate drugs by increasing the efflux of certain drugs, thereby decreasing drug efficacy [76]. P-gp has been found to be overexpressed in approximately 50% of all human cancers [77] and is known to play a crucial role in the development of multidrug resistance in cancer cells against anticancer drugs with distinct structures and mechanisms.

Both paclitaxel and docetaxel are good substrates of P-gp and able to increase expression of ABCB1 in many tumors and cancer cell lines. P-gp can restrict oral uptake of paclitaxel and facilitate elimination of the drug from the systemic circulation to the intestinal lumen in mice [78]. The Anticancer Drug Screen of the National Cancer Institute evaluated P-gp expression levels in a 60-cell line study and found a high correlation between expression of P-gp and cellular resistance to a large number of compounds, including paclitaxel [79]. Numerous studies have reported that overexpression of P-gp is involved in mediating resistance to paclitaxel and docetaxel in various types of cancers [80,81,82], including breast cancer. [83,84]. One study investigated the influence of expression of P-gp, multidrug resistance protein 1 (MRP1), and breast cancer resistance protein (BCRP) on the effectiveness of chemotherapy in patients with breast cancer. The results showed that in the absence of expression of transport proteins, an objective response (CR + PR) by the tumor was observed in 17 of 18 patients (94.4%). However, in the group with high membrane BCRP, MRP, and P-gp expression, 14 of 17 patients (82.3%) experienced disease progression [84]. Jin et al. reported that P-gp overexpression leads to shorter OS and DFS in 65 patients who receive neoadjuvant chemotherapy (paclitaxel + epirubicin) [85].

Other transporters have also been studied for their functions in tumor resistance to anticancer drugs. For example, MRP1 or ABCC1, was the first identified member of the ABCC subfamily, which belongs to the ABC transporter superfamily [86]. The expression of MRP1 provides resistance to several drugs. Overexpression of ABCC1 has been shown to be associated with resistance in many cancer types, including lung, breast, and prostate cancers [87]. ABCG2 (breast cancer resistance protein, BCRP) is the major drug efflux transporter in breast cancer. A number of studies have demonstrated that ABCG2 is associated with tumor chemoresistance in breast cancer [88,89]. The substrates of ABCG2 include chemotherapeutic drugs (mitoxantrone, bisantrene, epipodophyllotoxin, camptothecins, flavopiridol, and anthracyclines) and several TKIs (imatinib and gefitinib) [89,90]. However, other classes of anticancer drugs, including vinblastine, cisplatin, and paclitaxel, are not BCRP substrates [90]. In addition, ABCC3 can transport many chemotherapeutic drugs, and its overexpression results in multidrug resistance in breast cancer [91]. Similarly, ABCC10 is also related to drug resistance, and loss of ABCC10 can result in increased sensitivity to docetaxel treatment [92]. These findings suggest that other ABC transporters may have intricate functions in the efflux of taxanes. Therefore, it is important to consider these transporters when searching for targeted therapies.

### 2.4. Deregulation of Cell Death

Taxanes not only affect microtubule dynamics but also have different effects that result in cytotoxicity, inducing apoptosis in cancer cells through multiple signaling pathways [93]. Dysregulation of certain apoptotic pathways, including intrinsic and extrinsic pathways, has been found to be a key factor in the development of multidrug resistance in cancer cells [19]. Alterations in the intrinsic apoptotic pathway may be crucial for paclitaxel resistance [94]. The intrinsic pathway can be triggered by any stimulus that causes oxidative stress, mitochondrial disruption, and DNA damage, such as cancer therapeutic agents, hypoxia, and ischemia—reperfusion injury. Mitochondrial damage can lead to altered mitochondrial osmotic pressure and loss of transmembrane potential, prompting release of cytochrome C from the mitochondria into the cytoplasm, which then binds Apaf-1, thereby triggering the apoptotic cascade by activating procaspase 9 [95].

The Bcl-2 family of proteins are key regulators of the intrinsic pathway, including (a) apoptosis mediators (Bax, Bak, and Bok), (b) antiapoptotic proteins (Bcl-2, Bcl-XL, and Mcl-1), and (c) proapoptotic proteins (Bid, Bim, and Bad) [96,97]. It was demonstrated that treatment with microtubule-targeting agents, including taxanes, leads to an increase in posttranslational modification of the antiapoptotic Bcl-2 family [98,99]. Obviously, expression and function of the Bcl-2 family have a significant impact on taxane resistance [99,100]. As an antiapoptotic protein, Bcl-2 has been shown to inhibit cytotoxic chemotherapeutic agents that induce apoptosis through DNA damage or microtubule interference [101]. Overexpression of Bcl-2 can protect the 697 leukemia cell line from paclitaxel-induced apoptosis [102]. However, a previous study showed that overexpression of Bcl-2 increased sensitivity to both paclitaxel and vinorelbine in lung and breast cancer cells and significantly potentiated the in vivo efficacy of paclitaxel [98]. A possible explanation for the opposite effect is that there is a correlation between elevated Bcl-2 expression and increased expression of the pro-apoptotic protein Bim [98]. Similarly, inducing Bcl-2 overexpression in MCF-7 and MDA-MB-468 breast cancer cells by ABT-737, a Bcl-2 antagonist, can increase the efficacy of paclitaxel [94]. The aforementioned studies suggest that different tumors can impact the role of Bcl-2 protein in regulating the sensitivity of microtubule-targeted agent treatment.

Bcl-2-mediated taxane resistance can also be impacted by epidermal growth factors or hormones present in cells. For example, the growth factor signaling molecule insulin receptor substrate protein (IRS-1) can bind to the “loop region” of Bcl-2, inhibiting phosphorylation of Bcl-2 and leading to increased resistance to microtubule inhibitor drugs. In addition, it is well known that estrogen receptor (ER)-negative breast cancer is more sensitive to taxanes than ER-positive breast cancer, and one possible reason is that ER regulates Bcl-2-induced taxane resistance in breast cancer cells by inhibiting apoptotic cell death [103].

### 2.5. Alterations in Proliferation Signaling Pathways and EMT

Multiple signaling pathways regulate cell proliferation, and alterations in proliferation signaling pathways can lead to taxane resistance in cells. For instance, stimulation of the Akt signaling pathway is the crucial cause of taxane resistance. In a study that predicted a benefit from sequential addition of paclitaxel to adjuvant doxorubicin plus cyclophosphamide chemotherapy in patients with node-positive breast cancer participating in the National Surgical Adjuvant Breast and Bowel Project (NSABP) B-28 trial, results indicated that altered phosphorylation of Akt can contribute to resistance to paclitaxel but that patients with pAkt-negative breast tumors do not appear to benefit from addition of paclitaxel [104].

As a crucial mechanism for tumor metastasis, EMT also plays a role in resistance to chemotherapy. Several key signaling pathways, including TGF-β, Wnt, Notch, and Hedgehog, are known to be involved in EMT [105]. Paclitaxel-treated breast cancers display increased markers of TGF-β signaling, and TGF-β inhibition can enhance paclitaxel sensitivity against triple-negative breast cancer [106].

## 3. Long Noncoding RNAs

The transcription process involves production of both protein-coding messenger RNAs (mRNAs) and noncoding RNAs (ncRNAs) [107]. As a subclass of noncoding RNAs, lncRNAs consist of more than 200 nucleotides. Most lncRNAs have the same characteristics as mRNAs but are usually transcribed from fewer exons than coding RNAs and lack an open reading frame [108,109]. In general, lncRNAs are localized to the nucleus but can also be detected in the cytoplasm [110]. They are transcribed by RNA polymerase II, undergo 5′ capping, 3′ cleavage, and polyadenylation and produce mature lncRNAs through splicing, although the process is less efficient than that of mRNA splicing [108,111]. LncRNAs are typically classified into five categories based on their location relative to adjacent protein-coding genes, including intergenic lncRNAs (lincRNAs), antisense lncRNAs, sense lncRNAs, intronic lncRNAs, and bidirectional lncRNAs [112,113].

LncRNA expression is mainly tissue specific, suggesting that lncRNAs may play a functional role in physiological and biological processes [114]. Although the roles of most lncRNAs have not been verified, it has been shown that lncRNAs are involved in many areas of genome function, including epigenetics, gene transcription, splicing, and translation, as well as fundamental biological processes, such as cell cycle progression and differentiation [115]. In addition, lncRNAs perform cytoplasmic functions, mainly acting as miRNA sponges, regulating translation of specific mRNAs, and interacting with various signaling proteins [5].

**Figure 2:**
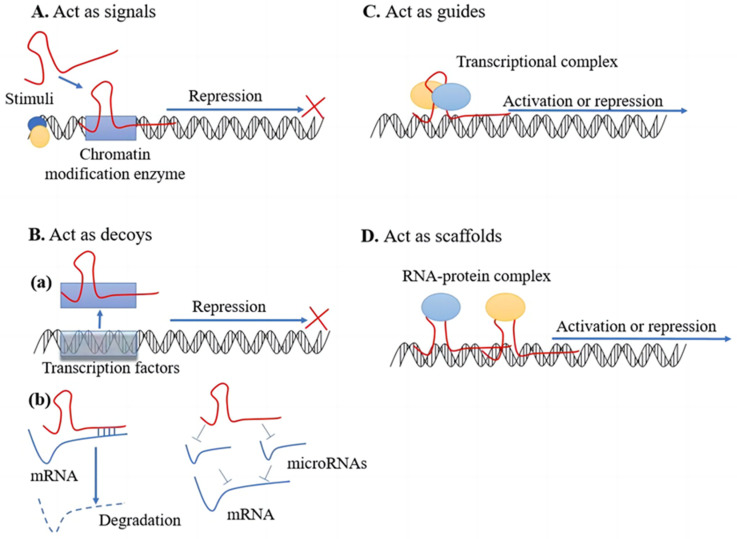
Mechanisms of action of lncRNAs. (**A**) Act as signals: Signaling lncRNAs respond to stimuli, receive signals, and interact with chromatin modification enzymes to prevent transcription. (**B**) Act as decoys: (**a**) Decoy lncRNAs have high affinity for selective transcription factors, and after binding to lncRNAs, the transcription process is inhibited because transcriptional regulators do not bind to DNA. (**b**) LncRNAs can also act as “sponges” for miRNAs, blocking the inhibitory effect of miRNAs on their downstream target mRNAs and thus regulating gene expression. (**C**) Act as guides: guide lncRNAs assemble transcription factors at specific loci and influence regulation of chromatin modification, thereby regulating transcription. (**D**) Act as scaffolds: Scaffold lncRNAs assemble RNA—protein complexes and promote or suppress transcription by suppressing or activating target genes.

Overall, lncRNAs are a group of diverse regulatory ncRNAs with different properties, locations, and mechanisms of action. The function of lncRNAs depends on their subcellular localization [113,116]. Several mechanisms of action have been proposed for lncRNAs (Figure 2).

The first mode of action involves signaling. LncRNAs are specifically transcribed under different stimuli and act as signaling molecules to participate in specific pathways, regulating the transcription of downstream genes. For example, lncRNA Kcnq1ot1 and Air mediate transcriptional silencing of multiple genes by interacting with chromatin and recruiting the chromatin-modifying machinery [117]. XIST, which mediates X chromosome inactivation in females, is also associated with this mechanism. During female development, Xist RNA is expressed from the inactive X chromosome and acts as a ‘coat’ on the chromosome it is transcribed from, leading to chromosome-wide repression of gene expression [118].

LncRNAs can also act as decoys and bind directly to selective transcription regulatory factors or RNA. As a result of lncRNA binding, transcription is repressed owing to lack of binding of transcription regulatory factors to DNA. For example, lncRNA PANDA can bind to NF-YA, a transcription factor responsible for the activation of apoptosis-related genes, resulting in suppression of its target genes [119]. Many lncRNAs also act as “sponges” for miRNA and block inhibition of miRNA on its downstream target mRNA, thereby indirectly regulating gene expression; examples are TUG1 and MEG3, which can isolate miRNA from mRNA and protein targets, altering protein translation [120]. Several studies have also revealed the lncRNA—mRNA interaction; lncRNAs that possess Alu elements can bind to the 3′UTR of mRNA and mediate mRNA degradation [121,122].

The third mode is guide-mediated action, in which lncRNAs guide chromatin modifiers or transcription factors to target genes and help them to localize appropriately at transcriptional loci, regulating transcription. It has been found that this transcriptional regulation by lncRNA mediators can occur through a cis- or trans-acting mechanism. An example of a cis-acting lncRNA is HOTTIP (HOXA Distal Transcript Antisense RNA), which promotes expression of the gene HOXA, and lncRNAs such as HOTAIR are able to alter and regulate epigenetic states in cells through targeting of the chromatin-modifying complex in trans [117].

In scaffold action, scaffold lncRNAs play an important structural role in assembling RNA—protein complexes, thereby promoting or suppressing transcription by activating or repressing target genes. For example, TINCR scaffolds the RNA-binding protein staufen1 with epidermal differentiation-promoting mRNAs that bind the TINCR box motif and promote its posttranscriptional stabilization. HOTAIR (HOX Transcript Antisense Intergenic RNA) scaffolds PRC2 proteins, thereby affecting chromatin accessibility and nuclear architecture and repressing transcription [123].

In general, lncRNAs regulate gene expression at three levels: epigenetic, transcriptional, and posttranscriptional regulation.

Epigenetic regulation is pretranscriptional regulation of eukaryotic gene expression, which mainly includes chromosomal architecture, histone modification, and DNA methylation and is an important part of gene expression regulation. LncRNAs usually combine with DNA, histone-modifying enzymes and transcription factors to participate in the pretranscriptional regulation of genes. As mentioned above, HOTAIR interacts with PRC2 to silence genes via scaffold action. Other lncRNAs, such as ANRIL, H19, and XIST, also repress gene transcription by recruiting protein complexes, such as histone modifiers and chromatin remodeling complexes [124].

Transcriptional regulation is the most important form of regulation of gene expression. LncRNAs can participate in the transcriptional regulation of target genes by regulating transcription of neighboring protein-coding genes, interacting with transcription factors, and forming triple helix complexes with DNA. LncRNAs can directly regulate gene expression by influencing the activity of enhancers [114]; lncRNAs themselves can also act as enhancer RNAs (eRNAs) to influence chromatin interactions [125].

In addition, lncRNAs can bind with proteins and mRNAs to form ribonucleoprotein complexes, which regulate posttranscriptional gene regulation. LncRNAs can also act as molecular decoys for miRNAs, sequestering miRNAs, and therefore inhibiting their regulatory effects on gene expression [126]. For example, lncRNA H19 has been shown to sponge and inhibit miR-675, suggesting a competing endogenous RNA (ceRNA) role for H19 lncRNA [127]. Furthermore, lncRNAs are involved in the translation machinery and regulate mRNA translation [113].

## 4. LncRNAs and Taxane Resistance

LncRNAs are recognized as important regulators of gene expression in cancer. Many lncRNAs have been implicated in cancer initiation and progression [108,128]. Abnormal expression of lncRNAs is closely related to tumor occurrence, metastasis, and tumor stage [129,130]. Furthermore, lncRNAs can directly or indirectly regulate a variety of pathways related to chemotherapy resistance, such as changes in drug efflux, inhibition of apoptosis pathways, and promotion of EMT [131,132,133].

As mentioned above, taxane resistance is a growing challenge in modern breast cancer chemotherapy, but the role of lncRNAs in mediating taxane resistance and susceptibility is not yet fully understood. However, several mechanisms are believed to be associated with taxane resistance induced by lncRNAs. First, ABC overexpression leads to enhanced drug efflux. ABC proteins, such as P-gp, ABCG2, and MRP, are frequently overexpressed in many types of cancers [134]. Multidrug resistance can be induced by overexpression of ABC efflux transporters due to specific lncRNAs [135]. A number of studies have shown that lncRNAs play a key role in increasing the outflow of a wide range of chemotherapeutic agents from a variety of cancer cells [136,137,138]. For example, UCA1 confers paclitaxel resistance through miR-129/ABCB1 axis in ovarian cancer [139]. In colorectal cancer, LINC00473 promotes paclitaxel resistance by activating the Bcl-2-related pathway and increases the LRP and MDR1 expression of MDR genes [140]. Second, many chemotherapeutic agents inhibit the proliferation of cancer cells by promoting apoptosis: lncRNAs associated to apoptotic pathways have been linked to multidrug resistance [132]. LncRNAs can protect cancer cells by inhibiting apoptosis. A previous study reported that after silencing LINC00511 expression, Bax and cleaved-caspase-3 increased with more cervical cancer cells arrested at the G1 phase [141]. In docetaxel-resistant lung adenocarcinoma cells, lncRNA CCAT1 was upregulated, and further study revealed that CCAT1 promotes chemoresistance by targeting the let-7c/Bcl-xl pathway [142]. Third, lncRNAs play a role in the EMT through signaling pathways, such as Wnt/β-Catenin and AKT/mTOR. Resistance/sensitivity to many chemotherapeutic agents has been associated with EMT-related lncRNAs [132]. In gastric cancer, lncRNA ZFAS1 can induce EMT via the Wnt/β-catenin pathway, thereby promoting chemotherapeutic tolerance [143]. 

**Figure 3:**
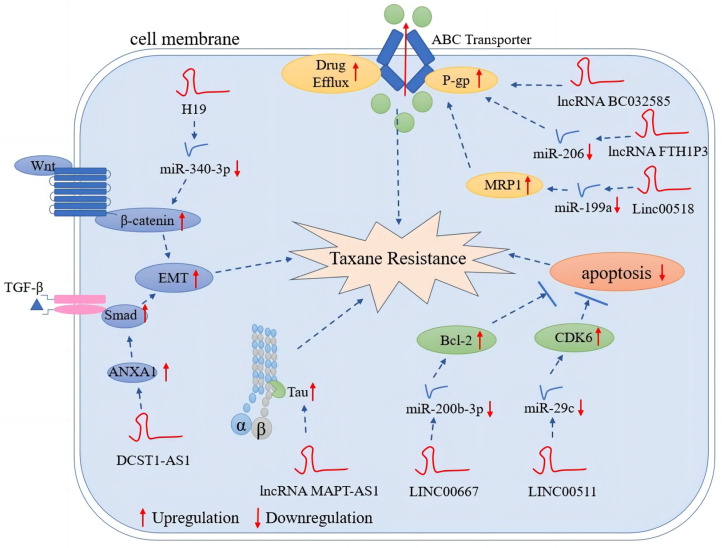
LncRNA-mediated mechanisms of taxane resistance in breast cancer. LncRNAs mediate taxane resistance by multiple molecular mechanisms, such as enhanced drug efflux, inhibition of apoptosis, promotion of proliferation signaling pathways and EMT, and alteration of MAPs.

Similarly, lncRNAs may mediate taxane resistance through these mechanisms in breast cancer (Figure 3). Studies of lncRNAs involved in taxane resistance in breast cancer are summarized in Table 1.

### 4.1. LncRNAs and Paclitaxel Resistance in Breast Cancer

As a microtubule-stabilizing agent, paclitaxel is an effective chemotherapy treatment option in clinical oncology. It causes cell cycle arrest at the G2 and M phases and induces apoptosis. In ER-negative breast cancer cells, lncRNA MAPT-AS1 was found to correlate with cell growth, invasiveness, and paclitaxel resistance through antisense pairing with the tau protein (MAPT) [144]. Some lncRNAs can mediate paclitaxel resistance by influencing ABC efflux transporters in breast cancer cells. For example, it has been reported that the lncRNA BC032585 might regulate MDR1 expression through either a cis- or trans-regulatory mechanism [145]. Knockdown of lncRNA BC032585 using specific siRNAs in MDA-MB-231 and MCF-7 breast cancer cells resulted in resistance to doxorubicin and doxorubicin plus paclitaxel treatment [145]. In contrast, the lncRNA FTH1P3 induced paclitaxel resistance in breast cancer cells by targeting the miR-206/ABCB1 axis to increase ABCB1 protein production [146]. Linc00518 was found to increase the resistance of MCF-7 cells to paclitaxel by reducing paclitaxel-induced apoptosis through the miR-199a/MRP1 axis, and linc00518 downregulation reduced multidrug resistance by regulating the miR-199a/MRP1 axis in breast cancer [147]. In paclitaxel-resistant MCF-7 cells, lncRNA RP11-770J1.3 and TMEM25 are highly expressed, and downregulation of lncRNA RP11-770J1.3 and TMEM25 can increase paclitaxel sensitivity in these cells by inhibiting expression of MRP, BCRP and P-gp [148].

In multiple studies, lncRNAs associated with the cell cycle and apoptosis pathways have been linked to taxane resistance in breast cancer. Linc00511 induces paclitaxel resistance in breast cancer cells by functioning as a ceRNA to sponge miR-29c and increase CDK6 expression [151]. Researchers found that lncRNA CASC2 expression was increased in paclitaxel-resistant clinical breast cancer samples and cell lines. Paclitaxel induces CASC2 expression in a concentration-dependent manner, and downregulation of CASC2 increases paclitaxel toxicity and decreases the IC50 value. The researchers further clarified that miR-18a-5p/CDK19 is the downstream target of CASC2 function [152]. Similarly, lncRNA UCA1 is highly expressed in paclitaxel-resistant breast cancer tissues and MCF-7 cells, and it was further confirmed that UCA1 mediates paclitaxel resistance via regulation of the miR-613/CDK12 axis [153]. Expression of the lncRNA NEAT1 is upregulated in cisplatin-resistant and paclitaxel-resistant MDA-MB-231 cells when compared with parental cells and knockdown of NEAT1-sensitized cells to chemotherapy. Functional studies have revealed that NEAT1 plays an oncogenic role by regulating apoptosis and cell cycle progression in triple-negative breast cancer (TNBC) cells [154]. LncRNA NONHSAT141924 was found to promote paclitaxel resistance by inhibiting the p-CREB/Bcl-2 apoptosis pathway, and overexpression of lncRNA NONHSAT141924 increases Bcl-2 and p-CREB protein levels in breast cancer [156]. Another study also showed that lncRNA H19 can reduce the sensitivity of breast cancer to paclitaxel by inactivating two key proapoptotic genes: BIK/NOXA [157].

In addition, lncRNAs can influence signaling pathways or EMT to regulate paclitaxel resistance in breast cancer. Some studies found that the level of lncRNA H19 expression in paclitaxel-resistant cells was significantly higher than that in paclitaxel-sensitive cells [161,162]. Knockdown of H19 lncRNA can restore chemosensitivity by mediating the AKT signaling pathway [161]. LncRNA DCST1-AS1 promotes TGF-β-induced EMT and enhances doxorubicin and paclitaxel resistance in TNBC cells through ANXA1 [164]. Similarly, linc-ROR was found to contribute to 5-fluorouracil and paclitaxel resistance and invasion of breast cancer cells by inducing EMT [165].

### 4.2. LncRNAs and Docetaxel Resistance in Breast Cancer

Docetaxel is a semisynthetic taxane that is structurally similar to paclitaxel and has the same or overlapping binding sites as paclitaxel. Docetaxel-derived tubulin polymers are structurally different from paclitaxel-derived tubulin polymers and have been shown to be more efficient in tubulin assembly due to their higher binding affinity for β-tubulin. Moreover, docetaxel remains in cancer cells longer than paclitaxel [172].

Although docetaxel has been shown to be effective in the treatment of breast cancer, resistance is often observed in patients. LncRNAs also play an important role in docetaxel resistance. Comprehensive RNA sequencing and analysis were performed on two docetaxel-resistant breast cancer cell lines (MCF7-RES and MDA-RES) and their docetaxel-sensitive parental cell lines. The results revealed that the lncRNA EPB41L4A-AS2 was expressed in the parental breast cancer cell lines but was absent in the docetaxel-resistant descendants; the decreased level of EPB41L4A-AS2 was also significantly associated with an increased level of ABCB1 mRNA [149]. In docetaxel-resistant breast cancer cells, the lncRNA LINC00680 expression level was upregulated, and LINC00680 was found to promote docetaxel resistance via the miR-320b/CDKL5 axis [158]. Similarly, the study found that the expression level of LINC00667 was significantly higher in docetaxel-resistant TNBC cell-derived exosomes than in docetaxel-sensitive TNBC cell-derived exosomes. Exosomal LINC00667 was found to promote the resistance of TNBC cells to docetaxel by regulating the miR-200b-3p/Bcl-2 axis [159]. Additionally, in breast cancer, LINC00461 can act as a ceRNA by directly interacting with miR-411-5p and promotes docetaxel resistance by acting as a sponge for miR-411-5p molecules [171]. LncRNA TMPO-AS1 has been identified as a prognostic indicator for patients with lung cancer. However, recent studies have shown that it can also contribute to docetaxel resistance and invasion in breast cancer by regulating the miR-1179/TRIM37 axis [167]. TRIM37 is an E3 ubiquitin ligase and is dysfunctional in a number of cancer types. TRIM37 enhances EMT in colorectal cancer [173] and activates the Wnt/β-catenin signaling pathway in hepatocellular carcinoma [174].

## 5. Conclusions and Perspectives

This review provides an overview of the main mechanisms underlying the development of taxane resistance in breast cancer and the role of lncRNAs in this process. The article discusses the basic functions of lncRNAs and presents comprehensive data and mechanisms on lncRNAs associated with taxane resistance in breast cancer, including regulatory roles of lncRNAs in microtubule-binding proteins, drug efflux pump metabolism, and the cell cycle and apoptosis regulation, as well as their impact on signaling pathways and EMT.

The relationship between lncRNAs and tumor development, including breast cancer, has been extensively researched. Many studies have established the significance of lncRNAs in taxane resistance in breast cancer, and advancements in next-generation sequencing technologies have provided better comprehension of the molecular changes that occur in drug-resistant tumors, thus enriching our understanding of drug resistance mechanisms. It is well established that lncRNAs participate in the development of taxane resistance via distinct mechanisms, including the regulation of drug efflux metabolism, suppression of cell death, enhancement of the proliferation signaling pathways, and EMT. Identification of the molecular mechanism of lncRNA’s role in taxane resistance in breast cancer may assist in the development of effective treatment modalities. Therefore, in addition to being used as early diagnostic and prognostic biomarkers for tumors, lncRNAs may have clinical uses as potential therapeutic targets for drug-resistant breast cancer. However, although silencing oncogenic lncRNAs may be an effective strategy to overcome taxane resistance, as a common problem in all forms of cancer gene therapy, lack of suitable and specific vectors for delivery is a major obstacle. So, more extensive and complete research about the biological effects of the lncRNAs are needed before using it as a real treatment strategy. Overall, a comprehensive and thorough understanding of the lncRNA-mediated mechanisms of taxane resistance in breast cancer is critical for clinical translation of future cancer treatments.

## Figures and Tables

**Table 1:** The function of lncRNAs in taxane resistance in breast cancers.

Mechanism of Action	lncRNA	Resistance Taxanes	Expression Level	Potential Targets	Reference
Regulate microtubule dynamics	MAPT-AS1	Paclitaxel	↑	↑MAPT	[144]
Regulate drug efflux metabolism	BC032585	Paclitaxel	↓	↑MDR1	[145]
	FTH1P3	Paclitaxel	↑	↓miR-206/↑P-gp	[146]
	Linc00518	Paclitaxel	↑	↓miR-199a/↑MRP1	[147]
	lncRNA RP11-770J1.3 and TMEM25	Paclitaxel	↑	↑MRP1, ↑BCRP1, ↑P-gp	[148]
	EPB41L4A-AS2	Docetaxel	↓	↑ABCB1 mRNA	[149]
Regulate apoptosis and the cell cycle	MA-linc1	Paclitaxel	↑	↓Purα	[150]
	LINC00511	Paclitaxel	↑	↓miR-29c/↑CDK6	[151]
	CASC2	Paclitaxel	↑	↓miR-18a-5p/↑CDK19	[152]
	UCA1	Paclitaxel	↑	↓miR-613/↑CDK12	[153]
	NEAT1	Paclitaxel	↑	-	[154]
		Paclitaxel	↑	↓miR-23a-3p/↑FOXA1	[155]
	NONHSAT141924	Paclitaxel	↑	↑p-CREB/Bcl-2	[156]
	H19	Paclitaxel	↑	↓BIK/NOXA	[157]
	LINC00680	Docetaxel	↑	↓miR-320b/↑CDKL5	[158]
	LINC00667	Docetaxel	↑	↓miR-200b-3p/↑Bcl-2	[159]
Regulate signaling pathways or EMT	LINC00160	Paclitaxel	↑	↑TFF3	[160]
	H19	Paclitaxel	↑	↑AKT signaling pathway	[161]
		Paclitaxel	↑	↓miR-340-3p/↑YWHAZ/Wnt/β-catenin signaling pathway	[162]
	OTDU6B-AS1	Paclitaxel	↑	↓miR-26a-5p/↑MTDH	[163]
	DCST1-AS1	Paclitaxel	↑	↑ANXA1/↑TGF-β-induced EMT	[164]
	ROR	Paclitaxel	↑	-	[165]
	AF178030.2	Paclitaxel	↑	↓TRPS1	[166]
	TMPO-AS1	Docetaxel	↑	↓miR-1179/↑TRIM37	[167]
Unclarified	HIF1A-AS2 andAK124454	Paclitaxel	↑	-	[168]
	DDX11-AS1	Paclitaxel	↑	↓miR-497	[169]
	SNHG7	Paclitaxel	↑	↓miR-34a	[170]
	LINC00461	Docetaxel	↑	↓miR-411-5p	[171]

↑ Upregulation; ↓ Downregulation.

## Data Availability

Not applicable.
